# *Pay attention: you can fall!* The Mini-BESTest scale and the turning duration of the TUG test provide valid balance measures in neurological patients: a prospective study with falls as the balance criterion

**DOI:** 10.3389/fneur.2023.1228302

**Published:** 2023-09-08

**Authors:** Antonio Caronni, Michela Picardi, Stefano Scarano, Chiara Malloggi, Peppino Tropea, Giulia Gilardone, Evdoxia Aristidou, Giuseppe Pintavalle, Valentina Redaelli, Paola Antoniotti, Massimo Corbo

**Affiliations:** ^1^Department of Neurorehabilitation Sciences, IRCCS Istituto Auxologico Italiano, Ospedale San Luca, Milan, Italy; ^2^Department of Biomedical Sciences for Health, University of Milan, Milan, Italy; ^3^Department of Neurorehabilitation Sciences, Casa di Cura Igea, Milan, Italy

**Keywords:** falling risk, neurological rehabilitation, psychometrics, criterion validity, gait assessment, balance assessment, inertial measurement unit

## Abstract

**Background:**

Balance, i.e., the ability not to fall, is often poor in neurological patients and this impairment increases their risk of falling. The Mini-Balance Evaluation System Test (Mini-BESTest), a rating scale, the Timed Up and Go (TUG) test, and gait measures are commonly used to quantify balance. This study assesses the criterion validity of these measures as balance measures.

**Methods:**

The probability of being a faller within nine months was used as the balance criterion. The Mini-BESTest, TUG (instrumented with inertial sensors), and walking test were administered before and after inpatient rehabilitation. Multiple and LASSO logistic regressions were used for the analysis. The diagnostic accuracy of the model was assessed with the area under the curve (AUC) of the receiver operating characteristic curve. Mobility measure validity was compared with the Akaike Information Criterion (AIC).

**Results:**

Two hundred and fourteen neurological patients (stroke, peripheral neuropathy, or parkinsonism) were recruited. In total, 82 patients fell at least once in the nine-month follow-up. The Mini-BESTest (AUC = 0.69; 95%CI: 0.62–0.76), the duration of the TUG turning phase (AUC = 0.69; 0.62–0.76), and other TUG measures were significant faller predictors in regression models. However, only the turning duration (AIC = 274.0) and Mini-BESTest (AIC = 276.1) substantially improved the prediction of a baseline model, which only included fall risk factors from the medical history (AIC = 281.7). The LASSO procedure selected gender, disease chronicity, urinary incontinence, the Mini-BESTest, and turning duration as optimal faller predictors.

**Conclusion:**

The TUG turning duration and the Mini-BESTest predict the chance of being a faller. Their criterion validity as balance measures in neurological patients is substantial.

## 1. Introduction

Falls are a leading cause of injury, disability, and injury-related death (1), and an increased risk of falling afflicts people with neurological impairments (2). Hence, balance, i.e., the ability not to fall (3), is paramount in medicine, particularly in neurology and rehabilitation.

Evaluating the patient’s stance, gait, and transferring ability is fundamental in assessing balance (1, 4). Once patients with poor balance are identified, treatments are available to reduce the balance impairment, eventually reducing their fall risk (5, 6).

Several rating scales have been developed for balance assessment. In these scales, clinicians rate the patient’s performance in different balance tasks. Scores are typically low if the patient cannot complete the item or when the chance of falling during testing is high (7).

The Mini-Balance Evaluation System Test (Mini-BESTest) (8) is a rating scale with high content and construct validity as a balance measure. Regarding the former, any clinician likely considers the Mini-BESTest items as good balance indicators. About the latter, the Mini-BESTest complies with the Rasch analysis requirements (9–12), thus returning high-quality, unidimensional balance measures.

Timed clinical and instrumental mobility tests are also used to assess balance.

Gait speed can predict several adverse events (including falls, hospitalization, and mortality), earning the “vital sign” title (13). An increased duration of the Timed Up and Go (TUG) test (14), in which the time a person takes to rise from a chair, walk a few meters, turn, walk back to the chair and sit down is measured, can indicate an increased risk of falls (1, 6).

In recent years, an instrumented version of the TUG test has been developed (i.e., the instrumented TUG, ITUG) (15) in which patients complete the TUG donning an inertial measurement unit. With these tools, many mobility measures can be obtained from the TUG test, with some of them, such as those from the turning phase, suitable for balance assessment (16–19).

Even if different rating scales, gait, and TUG test measures have shown validity as balance measures, there is still a real need to assess their criterion validity in greater detail. In this regard, studies using the risk of falling as the balance criterion standard seem particularly valuable.

The need for further investigations is particularly true for the ITUG measures, given the relatively young age of these devices. However, this also holds for well-referenced mobility tests since results have been inconsistent from study to study [e.g., (20)]. Moreover, the same measure can predict the risk of falling in a specific population but not in another (21).

The current work aims to assess if different mobility measures, including the Mini-BESTest, gait parameters, and measures from the ITUG, have satisfactory criterion validity as balance measures in neurological patients. The probability of becoming a faller, assessed prospectively within 9 months, was used as the balance criterion.

## 2. Methods

This is a longitudinal, prospective, observational study. From October 2018 to September 2020, participants were recruited among those admitted to the inpatient rehabilitation unit of Casa di Cura del Policlinico (Milan, Italy) because of a neurological disability. The local ethics committee approved the study (Comitato Etico Milano Area 2; 568_2018bis), and participants gave their written consent to participate. The current work reports the primary analysis of the project.

The study’s inclusion and exclusion criteria are listed below.

Inclusion criteria:

- Age > 18 years;
- Hemiparesis secondary to a stroke (ischaemic or haemorrhagic), peripheral neuropathy of the lower limbs, Parkinson’s disease, or vascular parkinsonism;
- Consent to participate in the study.

Exclusion criteria

- Concomitance of two neurological diagnoses (e.g., hemiparesis and Parkinson’s disease);
- The inability to complete the TUG test and the 10 m walking test without touching assistance on admission and discharge;
- A TUG duration longer than 30 s on discharge;
- Severe visual impairment or hearing loss;
- Rare neurological diseases.

The study only included stroke, peripheral neuropathies, and parkinsonism [and excluded rarer diseases also known to cause a balance and gait impairment, such as neuromuscular disorders (22)] since they represent three widespread and prototypical motor syndromes affecting gait and gross motor functions. In plain words, these three motor syndromes are common causes of increased risk of falling.

In detail, hemiparetic walking was represented by stroke patients, ataxic gait by peripheral neuropathies, and the gait disorder of the rigid-akinetic syndromes by Parkinson’s disease and vascular parkinsonism.

All hemiparetic patients included here had a clinical stroke diagnosis with brain imaging (i.e., CT scan or MRI) compatible with intracerebral hemorrhage or ischaemic stroke (23). All these participants suffered persisting focal neurological deficits (i.e., hemiparesis) at the time of their inclusion in the study (23).

The criteria by Postuma et al. (24) were used for diagnosing Parkinson’s disease. Vascular parkinsonism was diagnosed in case of significant signs of vascular encephalopathy in the brain CT or MRI scan associated with a clinical diagnosis of rigid-akinetic syndrome, i.e., in the case of parkinsonian features, presumably of vascular origin (25). Based on the above, patients with atypical, degenerative parkinsonism were excluded [e.g., (26)].

The peripheral neuropathy of the lower limb was diagnosed after a nerve conduction study. The peripheral neuropathy in the patients included here was axonal, sensory-motor, and length-dependent (18, 27). Diabetes was among the polyneuropathy most-frequent risk factors (18).

All patients completed 5 to 6 weeks of physiotherapy (two sessions/day, 45 min each, 5 days/week) and occupational therapy (one session/day, 45 min each, 3 days/week). The rehabilitation program followed recommendations for reducing the fall risk [e.g., (28); details can be found in (18)].

The study’s sample size was calculated as follows: in line with previous reports on the risk of falling in neurological patients [e.g., (29)], it was estimated that about 50% of participants would fall within 1 year. Based on this estimate, considering that each participant was followed up for 9 months and that the logistic regression was used for the primary analysis, we planned to recruit at least 213 patients. In this way, it was reasonable to expect at least 80 fallers in the nine-month study, a total number of cases that permits simultaneously assessing up to seven predictors in the logistic regression models (30). In estimating the maximum number of predictors simultaneously testable in a (logistic) regression model (here, seven plus the regression intercept), the “10 events per variable” rule of thumb was applied (31).

The STROBE checklist for reporting cohort studies was followed (32).

### 2.1. Falls recording

Falls, i.e., events “during which a person inadvertently comes to rest on the ground or other lower level” (1), were recorded 9 months after the rehabilitation discharge.

Participants received a monthly paper calendar and were asked to annotate on this calendar if a fall occurred and the day it happened (33). Moreover, research staff contacted all participants at the end of the first, second, third, sixth, and ninth months from discharge to maximize compliance.

Participants were classified into non-fallers, fallers, and recurrent fallers according to the number of falls they experienced in the observation period. In particular, fallers fell just once, and recurrent fallers were those with two or more falls in the follow-up period (34).

### 2.2. Participants’ gait and mobility testing

The Mini-BESTest (8), the 10 m walking test (35), and the ITUG (15) were administered to each participant at rehabilitation admission and discharge.

The three-meter variant of the TUG test (14) was performed here (turning point marked by a traffic cone) and recorded with an inertial measurement unit (mHT-mHealth Technologies, Bologna, Italy) secured to the participant’s back (15, 17, 18).

The Mini-BESTest balance measure (36) was expressed in logits (the higher, the better the balance), the measurement unit from the Rasch analysis.

The patient’s disability was measured with the Functional Independence Measure (FIM) (37) in both assessment sessions (motor and cognitive domains). Finally, additional participants’ information was collected on admission only, including age, gender, and diagnosis.

Details on the measures collected here are given in Supplementary appendix 1.

### 2.3. Statistical analysis

The criterion validity of the Mini-BESTest, gait, and ITUG measures was assessed by testing their ability to predict the probability of being a faller within 9 months after discharge. Multiple logistic regression and the Least Absolute Shrinkage and Selection Operator (LASSO) logistic regression were used.

Overall, 12 variables were assessed as potential faller predictors, including five features from the medical history:

1. age (years),
2. gender (male vs. female),
3. acute vs. chronic condition,
4. cognitive impairment (present vs. absent) and
5. urinary incontinence (present vs. absent).

Acute patients were those transferred to rehabilitation from an acute hospital. Chronic ones were admitted from the community (19). Cognitive impairment was diagnosed from the total score of the FIM cognitive domain (no impairment if the cognitive domain total score was ≥33). Urinary incontinence was also derived from the FIM scale (no incontinence if item 7 was ≥6).

Four gait and mobility measures were tested as predictors:

1. GS: gait speed (m/s),
2. WR: walk ratio (cm/number of steps/min),
3. MB: Mini-BESTest interval measure (logits) and
4. TUG: the total duration (s) of the TUG test.

Finally, three measures from the ITUG test were assessed:

1. STW: duration (s) of the sit-to-walk phase,
2. Turn: duration (s) of the first turning phase,
3. ω: peak angular velocity (°/s) along the vertical axis during the first turning phase.

All predictors, except variables 1–3 from the medical history, came from the discharge assessment.

#### 2.3.1. Criterion validity assessment

First, a multiple logistic regression model was arranged with variables 1–5 as predictors and faller status as the response variable. This model, nicknamed “h” since it only contains variables from medical history, was the reference model.

Next, balance and gait measures were added to model h. The following models, called “h+,” were evaluated:

1. h + GS, i.e., containing all the variables included in model h plus gait speed (GS);
2. h + WR;
3. h + MB;
4. h + TUG;
5. h + STW;
6. h + Turn;
7. h + ω.

A mobility measure had criterion validity if (i) it was a significant predictor of the faller status per the likelihood ratio test and (ii) the AIC of its h + model was smaller than the one of model h, with a difference >2 in absolute value (38).

The AIC difference was also calculated to compare the criterion validity of the different balance and gait measures. AIC differences <2 indicate that the two models are equally good. Differences >4 suggest that the model with the smallest AIC is sensibly better than the candidate model.

The diagnostic accuracy of model h and models h + was assessed by calculating the area under the curve (AUC) of the receiver operating characteristic (ROC) curve. The AUC’s 95% confidence intervals (95% CI) were calculated with bootstrap (10^4^ replicates).

LASSO logistic regression was used to investigate criterion validity further (39). The variables subdued to the LASSO regression were all the variables from model h plus the mobility measures from h + models whose AIC difference to model h was >2.

If the mobility measures were selected as predictors by the LASSO procedure, this was considered confirmatory evidence of criterion validity. Note that this analysis assessed gait and mobility measures simultaneously rather than separately, as done previously.

A secondary analysis was run with recurrent faller as the response variable (i.e., participants who have fallen at least twice vs. non-fallers or fallen only once). For sample size reasons, only the LASSO logistic regression was used for this analysis.

Finally, simple logistic regression was employed to provide reasonable cut-offs for the clinical application of the measures with good criterion validity. In detail, three probabilities of being a faller (i.e., 0.25, 0.50, and 0.75) are used to identify as many balance measure cut-offs and define four ranges of balance impairment. Patients whose balance measure (upper cut-off, say of the Mini-BESTest interval measure) is associated with a probability of being a faller <0.25 are considered to suffer a mild balance impairment. Those whose balance measure is associated with a faller probability between 0.25 and 0.50 are considered to have a moderate balance impairment. Finally, those with a severe and markedly severe balance impairment have a balance measure associated with a faller probability between 0.50 and 0.75 and >0.75, respectively. A sensitivity and specificity analysis was eventually calculated on the measurement cut-offs.

Like any regression, logistic regression is susceptible to extreme observations. In the current study, the duration of the turning and sit-to-walk phases presented some “far-outs” (40), and their distribution was right-skewed, similar to other TUG duration measures [e.g., (41, 42)]. Regarding the far-out observations, these were defined according to Tukey (40) as the observations more extreme than the third (or first) quartile plus (or minus) three times the interquartile range. Because of far-outs and skewness, turning and sit-to-walk durations were ln-transformed before entering the regression models, a solution that effectively worked out this statistical issue.

The median and the first to third quartile (1st–3rd Q) were used as central tendency and dispersion measures, respectively. The Wilcoxon test was used to test paired comparisons (e.g., change in the mobility measures before and after rehabilitation).

R version 4.2.0 was used for statistics and graphics. See Supplementary appendix 1 for details on the LASSO logistic regression.

## 3. Results

**Figure 1:**
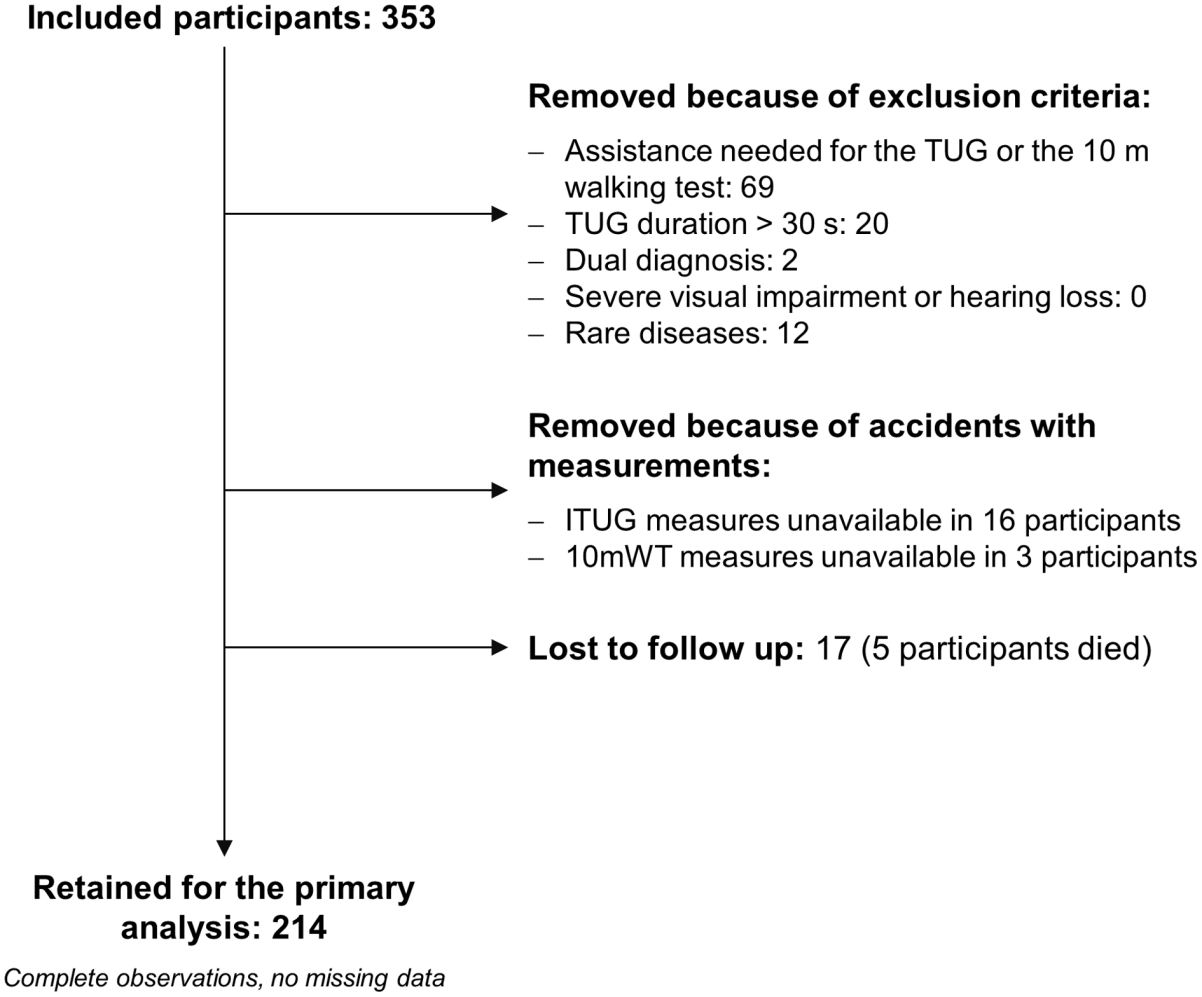
Study flow diagram. Regarding the two patients with a dual diagnosis, one had undefined peripheral neuropathy plus myopathy, and the second suffered hemiparesis due to a stroke and Parkinson’s disease. Eleven patients were affected by an uncommon polyneuropathy (e.g., chronic inflammatory demyelinating polyneuropathy, genetic demyelinating neuropathies), and one patient had a rare stroke (stroke in Behcet’s disease). Due to unexpected problems with the inertial sensors (e.g., the sensors were not fully charged), instrumented TUG (ITUG) measures were unavailable for 16 patients. Three patients were excluded because of an operator error during the 10 m walking test (10 mWT). The primary analysis consisted of logistic regression modeling with faller vs. non-faller as the response variable.

Of the 353 patients included in the study, 214 were retained for the primary analysis (Figure 1). Most participants (57.0%) had hemiparesis secondary to a stroke, followed by patients with peripheral neuropathy (24.3%; Table 1).

**Table 1:** Participants’ clinical characteristics.

Age, years, median (1st–3rd Q)		76.2 (66.7–81.2)	
Gender, *N* (%)	Males	124 (57.9)	
	Females	90 (42.1)	
Condition, *N* (%)	Acute	102 (47.7)	
	Chronic	112 (52.3)	
Diagnosis, *N* (%)	Hemiparesis	122 (57.0)	
	PNLL	52 (24.3)	
	PD	21 (9.8)	
	VP	19 (8.9)	
Cognitive impairment, *N* (%)	Present	80 (37.4)	
	Absent	134 (62.6)	
Assistive device, *N* (%)	Yes	101 (47.2)	
	No	113 (52.8)	
Urinary incontinence, *N* (%)	Present	33 (15.4)	
	Absent	181 (84.6)	
Motor FIM, score, median (1st–3rd Q)	Admission	67 (51–75)	*p* < 0.001
	Discharge	81 (75–86)	
Mini-BESTest, logits, median (1st–3rd Q)	Admission	0.17 (−0.53–1.32)	*p* < 0.001
	Discharge	1.32 (0.17–2.31)	
Gait speed, m/s, median (1st–3rd Q)	Admission	0.76 (0.58–1.02)	*p* < 0.001
	Discharge	0.96 (0.74–1.17)	
Step length, m, median (1st–3rd Q)	Admission	0.46 (0.38–0.55)	*p* < 0.001
	Discharge	0.50 (0.43–0.60)	
Cadence, steps/s, median (1st–3rd Q)	Admission	1.73 (1.52–1.93)	*p* < 0.001
	Discharge	1.90 (1.70–2.06)	
Walk ratio, cm/steps/min, median (1st–3rd Q)	Admission	0.46 (0.38–0.52)	*p* = 0.136
	Discharge	0.45 (0.39–0.52)	
Total TUG duration, s, median (1st–3rd Q)	Admission	17.3 (12.7–23.0)	*p* < 0.001
	Discharge	13.7 (10.9–17.9)	
Sit-to-Walk duration, s, median (1st–3rd Q)	Admission	1.32 (1.18–1.58)	*p* < 0.001
	Discharge	1.27 (1.13–1.48)	
Turn duration, s, median (1st–3rd Q)	Admission	3.12 (2.26–3.98)	*p* < 0.001
	Discharge	2.76 (2.16–3.34)	
Turn peak angular velocity, °/s, median (1st–3rd Q)	Admission	89.5 (71.6–114.5)	*p* < 0.001
	Discharge	104.6 (83.6–127.7)	

The FIM motor score and the mobility measures were collected on admission and discharge from inpatient rehabilitation. The presence of cognitive impairment, using an assistive device for independent walking, and urinary incontinence are from the evaluation at discharge. 1st–3rd Q: first to third quartile; N: number of *p*-values from the Wilcoxon signed rank test with continuity correction are in the rightmost column. The Wilcoxon test was used to assess a change in the mobility and disability measures between admission and discharge. Mini-BESTest measures are expressed in logits. As a total raw score, the median value of the Mini-BESTest measures corresponded to 14 (0.17 logits) at admission and 19 (1.32 logits) at discharge.

During the nine-month follow-up, 166 falls were recorded from 82 patients. Forty-two participants were recurrent fallers. Most falls caused no injury, while 38 were injurious falls. Of these, 25 caused a contusion, and seven a contused lacerated wound. Five resulted in a limb fracture, including a hip fracture, and one in a subdural haematoma.

A full description of the sample is given in Supplementary appendix 2.

### 3.1. Criterion validity analysis: fallers identification

The AUC of model h was 0.66 (95% CI: 0.59–0.74), pointing out some ability of this model to distinguish fallers from non-fallers. The AUC of h + models was also larger than 0.5 and negligibly higher than that of model h (Table 2).

**Table 2:** Validity analysis of the gait and mobility measures: multiple logistic regression.

Model	*b*	*e^b^*	*p*-values	AUC (95% CI)	AIC
h				0.66 (0.59–0.74)	281.7
Age, years	0.00	1.00			
Gender, male	−0.57	0.56			
Condition, chronic	0.76	2.15			
Cognitive impairment, yes	0.31	1.36			
Urinary incontinence, yes	0.65	1.92			
h + GS				0.67 (0.60–0.74)	280.2
Gait speed, m/s	−0.98	0.37	0.063		
h + WR				0.67 (0.59–0.74)	283.3
Walk ratio, cm/steps/min	0.97	2.64	0.565		
h + MB				0.69 (0.62–0.76)	276.1
Mini-BESTest, logits	−0.29	0.75	0.006		
h + TUG				0.68 (0.61–0.75)	279.4
TUG duration, s	0.06	1.06	0.040		
h + STW				0.67 (0.60–0.74)	280.4
STW duration, s	1.31	3.72	0.069		
h + Turn				0.69 (0.62–0.76)	274.0
Turn duration, s	1.45	4.24	0.002		
h + ω				0.68 (0.60–0.75)	278.5
Peak angular velocity, °/s	−0.01	0.99	0.023		

Coefficients (b) and exponentiated coefficients (e^b^) from the multiple logistic regression models. h: model h, the model only includes fall risk factors from the medical history. h+: models including the fall risk factors from the medical history (i.e., the same variables included in model h) plus a single gait or mobility measure. For example, in h + GS the gait speed (GS) is added to the risk factors from model h. WR, walk ratio; MB, Mini-BESTest; TUG, total duration of the TUG test; STW, sit-to-walk duration; Turn, TUG turning duration; ω, peak of the vertical angular velocity during turning. Only the estimate of the mobility measure is reported for the h + models. *p*-value: type 1 error probability of the likelihood ratio test comparing model h with one of the seven h + models. *p*-values < 0.05 indicate that adding the mobility measure to the fall risk factors from the history increases the model’s predictive accuracy. Therefore, the added mobility measure is a significant faller predictor. The area under the curve (AUC) and the Akaike information criterion (AIC) of the h and h + models are also reported. 95% CI: 95% confidence intervals of the AUC.

When h+ models were contrasted with model h (likelihood ratio test), the Mini-BESTest (*p* = 0.006), TUG (*p* = 0.040), and turning duration (*p* = 0.002) and the peak angular velocity during turning (*p* = 0.023) were significant faller predictors.

**Figure 2:**
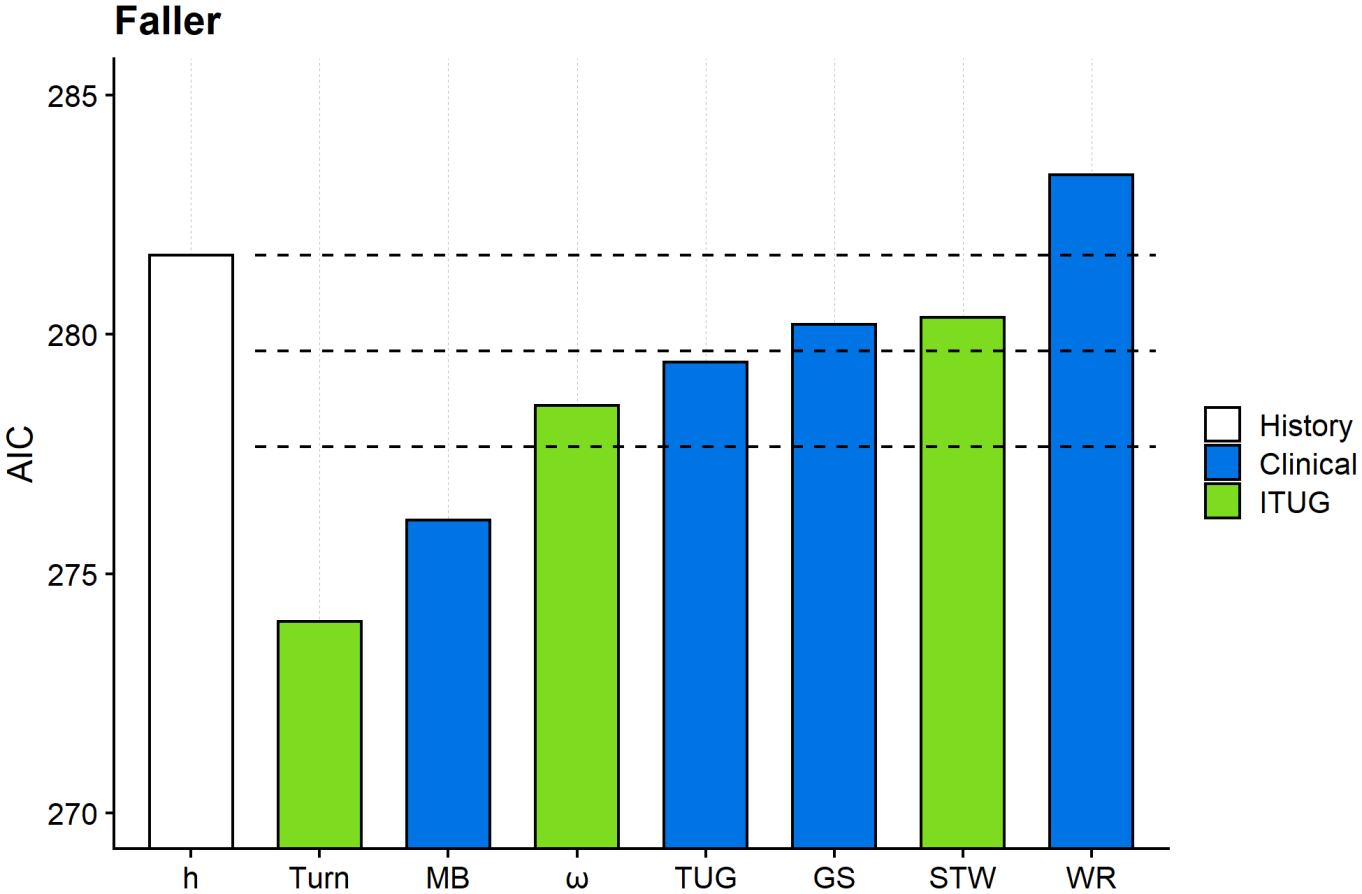
Comparing the criterion validity of the gait and mobility measures. The figure shows the Akaike information criterion (AIC) of model h and the seven h + models. Model h (white column) includes only fall risk factors from the medical history. For graphical reasons, Turn abbreviates model “h + Turn,” MB model “h + MB” and so on. Turn: duration of the TUG turning phase; MB: Mini-BESTest; ω: peak angular velocity along the vertical axis during the TUG turning phase; TUG: total TUG duration; GS: gait speed; STW: sit-to-walk duration; WR: walk ratio. Mobility measures from clinical tests and ITUG are given in blue and green, respectively. The uppermost horizontal dashed line marks the AIC of model h. The second and the third horizontal dashed lines mark −2 and −4 from the model h’s AIC. The response variable was the faller status (faller vs. non-faller) in all models.

Figure 2 shows the AIC of model h and h+ models. Considered altogether, significance testing and the AIC analysis indicated that turning duration and the Mini-BESTest were the mobility measures with the highest criterion validity for fall risk assessment.

**Figure 3:**
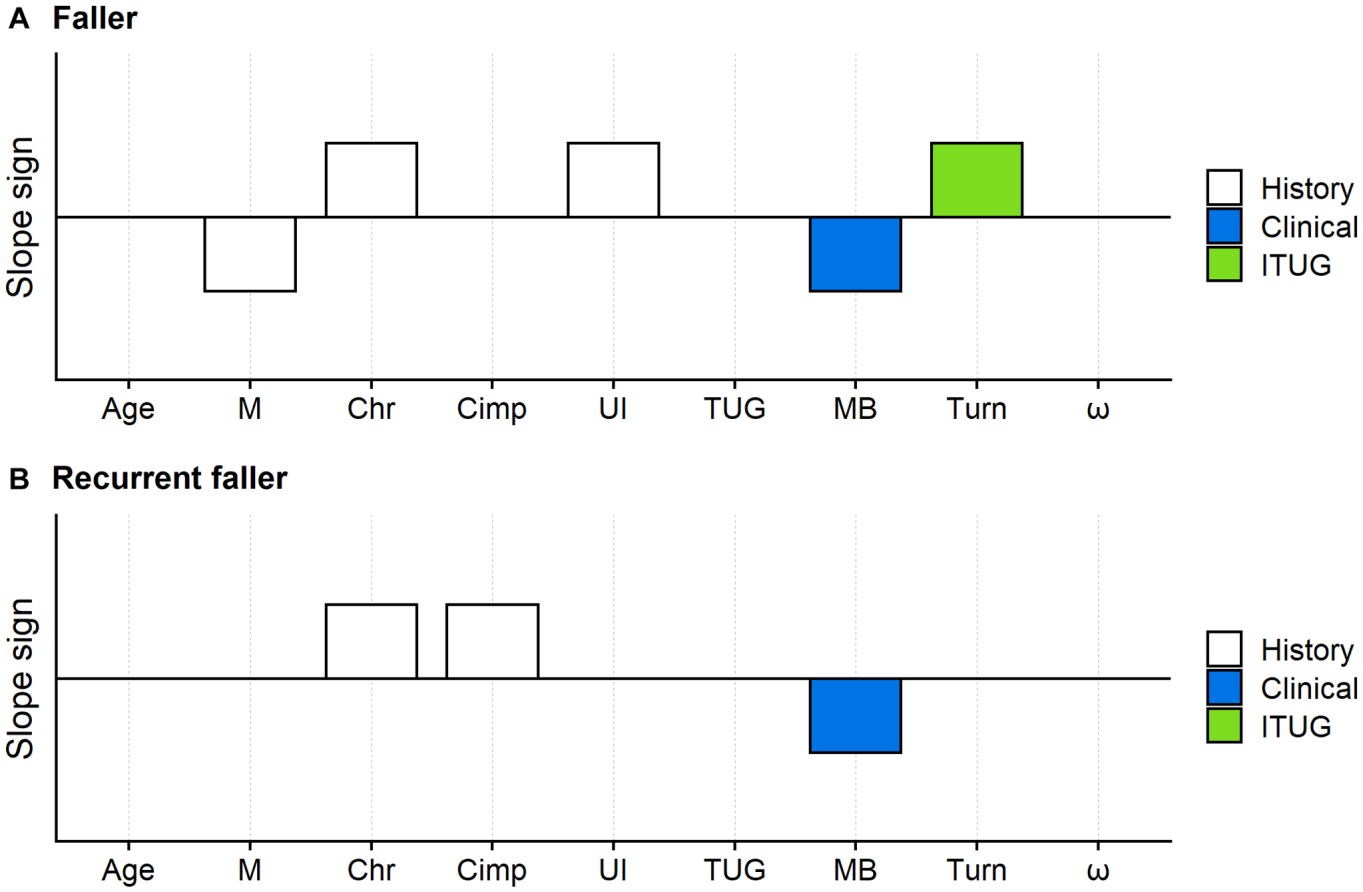
Faller and recurrent faller optimal predictors: results of the LASSO logistic regression. The variables simultaneously tested in the LASSO logistic regression models were: age, gender (M: male), condition (Chr: chronic disease), cognitive impairment (Cimp), urinary incontinence (UI), the TUG test total duration (TUG), the Mini-BESTest (MB) and the turn duration of the TUG test (Turn). White bars: fall risk factors from the medical history; blue bars: mobility measures from clinical tests; green bars: ITUG measures. The bars mark the predictors selected by the LASSO procedure, while variables without bars are those whose coefficients were shrunk to zero by the LASSO. Upward bars indicate positive predictors’ coefficients (i.e., variables positively associated with the faller or recurrent faller risk). Downward bars indicate otherwise. For example, M decreased the risk of being a faller, while Chr increased this risk. According to the LASSO logistic regression, the predictors of the optimal model for faller risk assessment **(A)** were: male gender, chronicity, urinary incontinence, the Mini-BESTest logit measure, and the turn duration. Optimal predictors for recurrent faller **(B)** were chronicity, cognitive impairment, and the Mini-BESTest.

The LASSO logistic regression substantially confirmed these findings. The Mini-BESTest measure and the turning duration were the only mobility measures selected as predictors of being a faller by the LASSO procedure, alongside gender, chronicity, and urinary incontinence (Figure 3A).

The AUC of this model was 0.69 (95%CI: 0.62–0.76).

According to the LASSO logistic regression, the chance of being a faller was higher for female patients than male patients, chronic than acute patients, and patients with urinary incontinence. Participants with low Mini-BESTest scores and high turn duration had a higher probability of becoming a faller.

### 3.2. Criterion validity analysis: identification of recurrent fallers

Figure 3B shows the results of the LASSO logistic regression with recurrent faller status as the response variable.

The LASSO procedure only selected chronicity, cognitive impairment, and the Mini-BESTest measure as optimal predictors (AUC: 0.71; 95%CI: 0.62–0.79).

### 3.3. Will my patient fall? Tentative cut-offs for fall risk assessment

**Figure 4:**
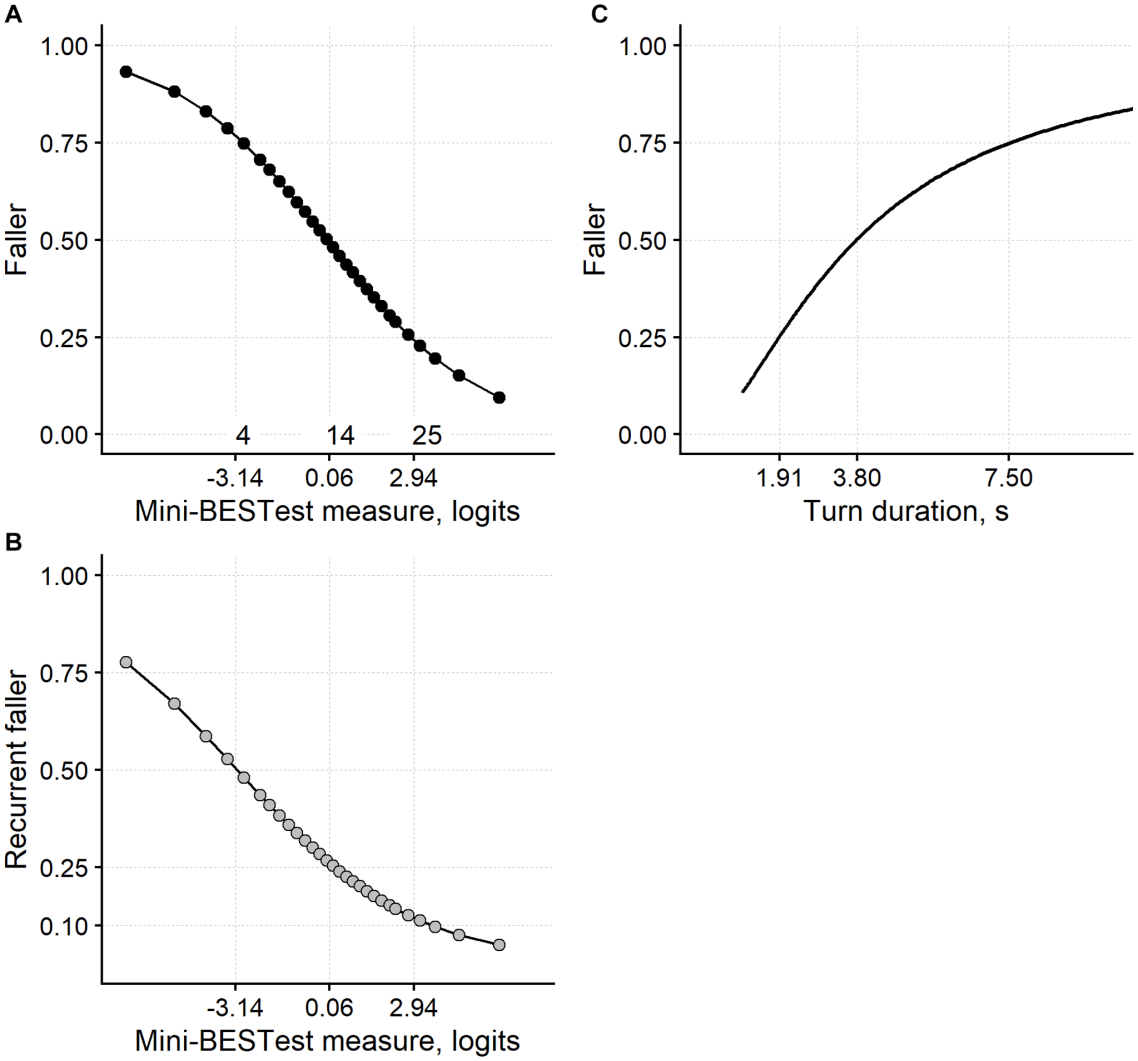
Fall risk assessment from the Mini-BESTest and the turn duration. **(A)** The relationship between the Mini-BESTest logit measure and the probability of being a faller within 9 months. **(B)** The relationship between the Mini-BESTest and the probability of being a recurrent faller. **(C)** The relationship between the turn duration and the faller probability. The curves are derived from simple logistic regression models. As expected from the main analyses, the Mini-BESTest has been confirmed as a significant predictor of the chance of being a faller (likelihood ratio test, *p* < 0.001) and a recurrent faller (*p* = 0.003). Turning duration significantly predicted the chance of being a faller (*p* < 0.001). Turn duration was ln-transformed for modeling and is plotted here back-transformed. Faller probability (0.25, 0.50, and 0.75) can be used to define four ranges of balance impairment delimited in the three plots by the vertical dashed lines. For example, it is proposed that a person measuring more than 2.94 logits on the Mini-BESTest (i.e., scoring ≥25) suffers a mild balance impairment since their risk of falling is <0.25. A person measuring between 0.06 and 2.94 logits suffers a moderate balance impairment and so on for a severe and very severe balance deficit. The same reasoning applies to the turn duration. The numerals “4,” “14,” and “25” in panel **(A)** correspond to the Mini-BESTest total ordinal score immediately above the logit thresholds.

Figures 4A,B show the relationship, from simple logistic regression, between the Mini-BESTest measure and the probability of being a faller and a recurrent faller, respectively. For application in the clinic, the probability of being a faller was used to split the Mini-BESTest measures into four ranges, defining four levels of balance deficit.

Figure 4C shows the relationship between the (back-transformed) duration of the TUG turning phase and the probability of being a faller. Again, four levels of balance impairment were distinguished from faller probability.

The Mini-BESTest cut-off separating mild and moderate balance deficit (i.e., 2.94 logits; the test is positive if the Mini-BESTest measure is below this threshold) had a sensitivity of 0.92 (specificity: 0.20; Table 3) for faller identification, thus identifying with a good approximation those subjects less at risk of falling due to a balance disorder. The 0.06 logits cut-off, which marks the limit between a moderate and severe balance deficit, had a specificity of 0.86 (sensitivity: 0.29).

**Table 3:** Sensitivity and specificity analysis of the cut-offs proposed for fall risk assessment.

MB			Turning duration		
***Cut-off: 0.06 logits (14)***			***Cut-off: 3.80 s***		
	**Fallers**	**Non-fallers**		**Fallers**	**Non-fallers**
Positives	24	18	Positives	20	17
Negatives	58	114	Negatives	62	115
Sn: 0.29; Sp: 0.86			Sn: 0.24; Sp: 0.87		
***Cut-off: 2.94 logits (25)***			***Cut-off: 1.91 s***		
	**Fallers**	**Non-fallers**		**Fallers**	**Non-fallers**
Positives	75	106	Positives	79	111
Negatives	7	26	Negatives	3	21
Sn: 0.92; Sp: 0.20			Sn: 0.96; Sp: 0.16		

MB, Mini-BESTest scale; Sn, sensitivity; Sp, specificity. For the MB scale, the cut-offs are provided as interval measures expressed in logits and total ordinal scores (in brackets). Turning duration cut-offs are provided back-transformed as in Figure 4. Participants are classified as fallers or non-fallers and as positive or negative to tests. Hence, fallers testing positive are true positives, non-fallers testing negative are true negatives, fallers testing negative are false negatives, and non-fallers testing positive are false positives. The MB scale and the Turning duration are used as tests to detect the patient who will fall, i.e., the test is considered positive when the measure flags a person as a future faller. Therefore, the MB is positive if its measure (or ordinal score) is below the cut-off value. The turning duration is positive if above the cut-off.

For the turning duration, the cut-off between a mild and moderate balance impairment (1.91 s, back-transformed; the test is positive if the turning duration is above this threshold) had a sensitivity of 0.96 (specificity: 0.16). The 3.80 s cut-off had a specificity of 0.87 (sensitivity: 0.24; Table 3).

## 4. Discussion

The current study shows that the Mini-BESTest scale and the duration of the turning phase of the TUG test measured with an inertial measurement unit have substantial criterion validity as balance measures.

Both these measures predict the patient’s probability of being a faller within 9 months. In addition, the Mini-BESTest (but not the turn duration) is also valid for predicting the chance of being a recurrent faller.

Assessing criterion validity involves assessing the degree to which the measures adequately reflect those from a criterion standard (43). In the current study, fall risk is supposed to be the criterion standard for balance.

Relating balance directly to falls is well-aligned with the balance construct definition and with previous studies evaluating the criterion validity of balance measures.

Balance has been defined as a person’s ability not to fall (3). Therefore, a fall indicates, by definition, that a person has a decreased “ability not to fall” and hence a poor balance. Furthermore, when a person is about to fall during a motor task, it indicates that the “ability not to fall” is reduced. In this regard, the lowest balance level is indicated in some items of the Performance Oriented Mobility Assessment – Balance (POMAB), the Berg balance scale, and the Mini-BESTest (likely the most used balance scales) by a fall or a near-fall (7).

Finally, the current work aligns with studies in which the criterion validity of other balance tests has been evaluated by testing their ability to predict falls [e.g., (33, 44)]. Some authors have stated that a measure of balance that identifies individuals prone to falling has predictive (i.e., criterion) validity (45).

Supplementary appendix 3 reports an excursus on the validity of mobility measures when used as balance measures.

### 4.1. Balance measures in the clinic: predicting the risk of falling

The ability to predict falls of some of the mobility measures evaluated here has already been studied (21), meaning it seems essential to clarify what our work adds to the present state of knowledge.

First, only neurological patients with mobility impairment have been recruited here. While deeply investigated in community-dwelling (including independent) participants of older age, falls are less studied in more severely impaired patients (5).

Moreover, unsurprisingly, a test that works well in community-dwelling, independent persons may not work well when applied to disabled individuals. For example, the TUG test does not discriminate fallers from non-fallers in high-functioning elderly. On the contrary, it is more valuable in lower-functioning older people (21).

Patients suffering from a neurological disorder, like those studied here, are “by definition” at an increased risk of falling (2). However, in the clinic, it is crucial to discriminate the patients at a very high risk of falling from those at a relatively lower risk. This practical question prompted the current study.

The definition of the four levels of balance impairment (Figure 4) and the sensitivity and specificity analysis of meaningful cut-offs for the Mini-BESTest and the turning duration try to answer this question.

For both the Mini-BESTest and the turn duration, the cut-off that demarcates a mild from a moderate balance impairment has a high sensitivity (>0.90) and poor specificity for identifying a faller. In contrast, the cut-off between moderate and severe balance deficit has high specificity (>0.85) but low sensitivity. Therefore, these cut-offs could work as “SnOUT” and “SpIN” tests, respectively (46).

In addition, these cut-offs could be of great interest for setting therapeutic goals. For example, improving the patients’ balance above 2.94 Mini-BESTest logits through rehabilitation could be considered a clinically important goal since falling is substantially less probable beyond this threshold.

The same reasoning applies to the 1.91 s turning duration threshold.

The current study is not the first to evaluate the Mini-BESTest ability to predict falls. However, in several studies, falls have been collected retrospectively [e.g., (47–50)]. The results of our study align well with other studies in which the Mini-BESTest was anchored to prospective falls, confirming this scale has a sensible capacity in fall risk assessment [e.g., (51)].

A novelty of our work is that the ability in fall risk evaluation of measurements from inertial sensors, like the Turning duration, has also been considered. Research that assesses the risk of falling from movement measures obtained with these devices is still a young field. For example, a recent meta-analysis showed that using sensor measures during walking and sit-to-stand actions can discriminate between fallers and non-fallers but the same meta-analysis concluded that their discrimination accuracy remains undetermined (52).

In addition, it should be stressed that similar to the Mini-BESTest, retrospective (i.e., history of falls) rather than prospective falls (as done here) are often used as the criterion standard (53). In this regard, it is noteworthy that using fall history as the standard for classification has been criticized by some scholars in fall risk assessment studies (54).

Finally, the findings concerning the walk ratio are noteworthy as among the seven gait and mobility measures, the walk ratio performed worse in terms of faller risk assessment. As with any negative result, we feel that more research is needed before concluding that the walk ratio has no criterion validity for balance assessment.

The walk ratio could work in fall risk assessments when other versions of the walking test are administered [e.g., fast walking (55)]. Interestingly, and potentially in line with the current findings, it has been shown that a reduced walk ratio is associated with fall risk in people with high gait speed (>1.0 m/s) but not in impaired persons walking at a lower speed (<1.0 m/s) (56).

A peculiar feature of the walk ratio is that it is constant at different walking speeds, indicating that gait speed is increased by raising both step length and cadence. Therefore, when the walk ratio decreases, which is generally the case here, an actual motor control law is violated.

Although disappointing, that a parameter with strong physiological and pathophysiological validity, such as the walk ratio, does not work well in the clinic is not an unusual finding. Regarding the fall risk assessment, this seems to be the case with dynamic posturography. In dynamic posturography, patients stand on moving platforms: the face validity of this task for assessing balance is unquestionable. However, surprisingly enough, dynamic posturography could not predict falls (57).

Not only the walk ratio but also gait speed performed poorly in the current study. This is an unexpected finding (58), given the recommendations of some authoritative studies (1, 2). In summary, gait parameters seemed to perform less well than other mobility measures in fall risk assessment.

### 4.2. Study’s limitations and future developments

First, the diagnostic accuracy of the models tested here is limited. Even if the AUCs of the ROC curves are significantly larger than 0.5, these are 0.7 at most. However, these AUC values align with previous reports on fall risk assessment [e.g., (21, 33, 59)].

Caveats should be put forward regarding the sensitivity and specificity analysis of the Mini-BESTest and turn duration cut-offs. Tests with high sensitivity but insufficient specificity could work suboptimally to “rule out” a condition. The same applies when tests with high specificity, but reduced sensitivity, are used to “rule in” a condition (46). Altogether, these facts strengthen the idea that the current work should be considered a metrology study about validity rather than a diagnostic one.

As reported in the Methods section, the diseases included in the sample represented three primary motor syndromes: hemiparesis, parkinsonism, and sensory ataxia. This classification is reasonable for syndrome-level disciplines such as Physical and Rehabilitation Medicine in the first place. However, we have to admit that it could be limited for the neurologist.

Future development of this line of research could consider the patients’ neurological profiling in greater detail. In this context, it is also noteworthy that disease-specific scales such as the National Institutes of Health Stroke Scale (NIHSS) or the Unified Parkinson’s Disease Rating Scale (UPDRS) could have a fall risk prediction value that has not been considered here.

We also feel it important to stress that while we generally refer here to fall risk assessment in “neurological patients,” our findings apply only to the three motor syndromes studied here. Future works are needed to assess the falling risk in rare and selected neurological diseases (e.g., demyelinating polyneuropathies, atypical parkinsonisms, neuromuscular disorders).

Regarding the patients recruited, we excluded those needing more than 30 s to complete the TUG test. However, in some patients, the TUG test duration can be longer, with studies reporting TUG test durations of two or more minutes [e.g., (60)]. Taking this into account, the current work did not consider those persons with a motor impairment of extreme severity, which could be those with the highest falling risk.

Clinical and methodological reasons prompted the approach used in this study. First, it is obvious that a TUG test duration >30 s already flags a clinically severe mobility impairment. In this regard, it should be stressed that 30 s is approximately three times the upper limit of the “healthy” 3 m TUG test duration (60). As rehabilitation clinicians, we believe that when the gait and balance impairment is so severe, there is likely little added value from a timed or even instrumented test. In these cases, scales, even relatively simple and classical ones such as the Performance Oriented Mobility Assessment’s balance domain (61), can fruitfully serve the job.

From a methodological point of view, software algorithms have been used here to automatically split the TUG test into different phases and obtain measures from them. While the dependability of these algorithms has been demonstrated (15), it should also be noted that automatic algorithms can fail, for example, in selected populations such as frail individuals (62). In this regard, it seems reasonable that the more gait and mobility are pathological (e.g., the slower the patient), the more challenging it will be for the algorithms to recognize mobility patterns and thresholds for TUG test segmentation.

As another methodological point, defining no upper limitation to the TUG test duration would allow the inclusion of persons with a TUG test duration of 60 s or even longer. Even when participants suffering from a severe mobility impairment can be found (60), they are likely a minority. In addition, people who can walk without physical assistance (see the second exclusion criterion) with such a long TUG test duration would be even rarer. Including these persons would increase the chance of including far-outs in the dataset, a statistical issue carefully considered in the primary analysis (see the Methods section).

As reported above, the fact that gait speed does not predict falls is an unexpected finding given its importance in the patients’ assessment (13). In a sense, gait speed is an omnibus measure since it is the product of step length and step cadence (i.e., step frequency). It can be hypothesized that if even just one of these two component measures is corrupted by a significant measurement error (63), gait speed accuracy in fall risk evaluation would also be compromised. Based on this consideration and the results reported here, assessing the criterion validity in fall prediction and balance assessment of step length and step cadence separately is a reasonable continuation of the current line of research.

### 4.3. Conclusion

The conclusions of this study are as follows: (i) the Mini-BESTest scale and the turn duration of the TUG test predict the probability of a neurological patient falling at least once in 9 months; (ii) the Mini-BESTest, but not the turn duration, also predicts the patient’s probability of becoming a recurrent faller (i.e., falling two or more times); and (iii) the criterion validity of the TUG turning duration as a balance measure is high, and that of the Mini-BESTest is even higher.

Adopting the “seeing to foresee, foreseeing to provide” motto, correctly predicting the risk of falling allows adequate fall prevention through information on behaviour and pharmacological and non-pharmacological interventions. In this line of reasoning, an obvious next step in the current line of research is developing algorithms simultaneously including all the relevant variables for fall risk assessment to obtain an instrument to accurately define the risk of falling at a single-person level.

## Data availability statement

The raw data supporting the conclusions of this article will be made available by the authors, without undue reservation.

## Ethics statement

The studies involving humans were approved by Comitato Etico Milano Area 2, Milan, Italy. The studies were conducted in accordance with the local legislation and institutional requirements. The participants provided their written informed consent to participate in this study.

## Author contributions

AC and MC: study conception. MP, EA, GP, VR, and PA: data collection. MP, GG, PA, and CM: database organization. AC: statistical analysis, figures’ preparation, writing the first draft of the manuscript, and manuscript review according to the co-author modifications. MP, SS, CM, PT, GG, EA, GP, VR, PA, and MC: manuscript commenting. All authors contributed to the article and approved the submitted version.

## Funding

The in-house resources of the Casa di Cura del Policlinico Spa supported the project and data collection. The research was also funded by the Italian Ministry of Health – Ricerca Corrente (IRCCS Istituto Auxologico Italiano, RESET project, 24C822_2018).

## Conflict of interest

The authors declare that the research was conducted in the absence of any commercial or financial relationships that could be construed as a potential conflict of interest.

## Publisher’s note

All claims expressed in this article are solely those of the authors and do not necessarily represent those of their affiliated organizations, or those of the publisher, the editors and the reviewers. Any product that may be evaluated in this article, or claim that may be made by its manufacturer, is not guaranteed or endorsed by the publisher.

## Supplementary material

The Supplementary material for this article can be found online at: https://www.frontiersin.org/articles/10.3389/fneur.2023.1228302/full#supplementary-material
