# Supplementary material for: Pay attention: you can fall! The Mini-BESTest scale and the turning duration of the TUG test provide valid balance measures in neurological patients: a prospective study with falls as the balance criterion
Source: Front Neurol. 2023 Sep 8;14:1228302. doi: 10.3389/fneur.2023.1228302 (PMC10516579; doi:10.3389/fneur.2023.1228302)
Supplement: Supplementary file 1 [file Data_Sheet_1.docx]

Supplementary Material

*Pay attention: you can fall!* The Mini-BESTest scale and the turning duration of the TUG test provide valid balance measures in neurological patients: a prospective study with falls as the balance criterion.

Antonio Caronni*, Michela Picardi, Stefano Scarano, Chiara Malloggi, Peppino Tropea, Giulia Gilardone, Evdoxia Aristidou, Giuseppe Pintavalle, Valentina Redaelli, Paola Antoniotti, Massimo Corbo

*** Correspondence:** Antonio Caronni, [a.caronni@auxologico.it](mailto:a.caronni@auxologico.it)

The following pages detail the methods and the results reported in the study's main text.

This Supplementary Material is organised in notes nested in three appendices. Each note develops and treats a specific point from the main manuscript and can be read independently from the others.

# Supplementary Methods

## Balance, gait and mobility testing

The participants' walking and balance abilities were assessed with the 10 m walking test, the Mini-BESTest and the TUG test. All tests were performed at rehabilitation admission and discharge. In addition, the patient's disability was measured with the Functional Independence Measure (FIM), motor and cognitive domains, in both assessment sessions. Additional participants' information was collected on admission only, including age, gender, diagnosis and if they had an acute or chronic condition.

The Mini-BESTest (1,2) is a 14-items, clinician-administered scale. Each item is scored in three categories, labelled 0, 1 and 2, according to the participant's ability to complete balance tasks. The total scale score ranges from 0 to 28 (the higher, the better).

The 14 items are grouped into four domains, each investigating a different balance aspect from static balance (i.e. the sensory orientation domain) to balance during walking (i.e. the dynamic gait domain). Representative items are item 8 "standing with feet together and eyes closed on a foam surface" (sensory orientation domain), and item 11 "walking with head turns" (dynamic gait domain).

Developed with the Rasch analysis (3–5), the Mini-BESTest ordinal scores can be easily turned into interval measures with logits as measurement units. What matters for practical use is that logits can be easily conceived as Celsius or Fahrenheit degrees: they can be positive or negative and usually range around 0 logits. Regarding the Mini-BESTest, the larger a person's logit measure, the higher the person's probability of passing a Mini-BESTest item. Hence: the higher the logit measure, the better the balance.

The FIM scale (6) returns disability measures with high content, construct and criterion validity. This clinician-administered scale comprises 18 items, split into a motor (13 items) and a cognitive (5 items) domain. FIM items test a person's ability to complete activities of daily living, such as eating and transferring (motor domain) and interacting with others (cognitive domain). FIM items are scored in seven categories (from 1 to 7). Persons scoring 1 need full assistance to complete a task, while those scoring 7 complete the task autonomously.

In the 10 m walking test (7), patients are asked to walk straight while the time spent travelling the central six meters of a 10 m linear trajectory is measured with a stopwatch. The steps used to travel these six meters are also counted. From these measures, the mean gait speed (m/s) and step length (m) and step cadence (steps/s) are calculated.

The walk ratio (8), i.e. the ratio between the step length and the step cadence (cm/steps/min), is a gait parameter deemed to reflect the nervous control of the gait cycle, as well as the energy expenditure and balance during walking (8). The walk ratio (expected value: about 0.65 cm/steps/min) is typically reduced in diseases such as Parkinson's disease, characterised by a decreased step length and increased step cadence.

The three-meter variant of the TUG test (9) was performed here. Prompted by a go signal from the experiment, participants got out of a chair, walked three meters, turned around, walked back to the chair and sat down. The turning point was marked with a traffic cone. The TUG duration (i.e. the time from the go signal to the patient sitting down on the chair) was measured with a stopwatch.

The participants completed the TUG test with an inertial measurement unit (mHT-mHealth Technologies, Bologna, Italy) secured to their back, approximately at the height of the 2^nd^ -3^rd^ lumbar vertebra. For technical details on the inertial measurement unit, see supplemental materials 1 in (10). Signals from the inertial sensors were used to split the TUG test into four phases (sit-to-walk, walking, turning and turn-and-sit) using validated algorithms (11,12).

## Details on the LASSO logistic regression for validity assessment

The LASSO regression was used in this study to complete the validity assessment.

The LASSO regression is a type of regression with shrinkage. The core of the LASSO regression is the LASSO procedure which minimises the model's residual sum of squares inflated by a quantity (13). Because of this added quantity (a penalty, actually), some regression coefficients tend to be precisely zero (13), so the corresponding predictors are dropped from the model (14).

LASSO regression was preferred to multiple logistic regression for the last steps of the analysis for sample size reasons and because it handles well in the case of multicollinearity (while multiple regression does not), which is typical for mobility measures (15).

Regarding the LASSO procedure run here, the tuning parameter λ was chosen with cross-validation, as customary (14). Because of the moderate sample size of the current study, it was possible to use leave-one-out cross-validation.

The "glmnet" library (16) was used for calculating the LASSO logistic regression.

# Supplementary Results

## An in-depth description of the patient's sample

Overall, on discharge from rehabilitation, the patient's sample suffered a mild to moderate balance impairment, as pointed out by the median Mini-BESTest measure (1.32 logits; 1^st^ to 3^rd^ quartile: 0.17 to 2.31logits), which corresponded to a median total score of 19 (out of 28). In addition, the discharge gait speed was reduced (0.96 m/s; 0.74 to 1.17 m/s), and patients suffered mild disability (FIM motor scale score: 81; 75 to 86).

Compared with the admission assessment (detailed in Table 1 in the main text), on discharge, patients significantly improved their Mini-BESTest measure, gait speed, total duration of the TUG test, sit-to-walk and turn duration, and turn peak angular velocity (Wilcoxon signed rank test with continuity correction: p < 0.001). On the contrary, no modification of the walk ratio was found (main text Table 1).

Regarding the faller status, seventeen participants could not be classified as faller or non-faller since they were lost at follow-up. Of these, five persons died. An additional person died between the sixth and ninth-month follow-up. However, this participant was included in the primary analysis since his faller status was known (he had fallen between months three and six). No death was related to a fall.

# Assessing the validity of mobility measures as balance measures.

## Criterion validity and the balance construct

Criterion validity, one of the three pillars of validity, with content and construct validity, reflects the degree to which the measures under evaluation adequately reflect those from a criterion standard (17).

Criterion validity is further split into concurrent and predictive validity. To be exact, since the present study has evaluated the ability of these measures to predict the future faller status, predictive criterion validity has been assessed.

Clearly, criterion validity can only be assessed when a criterion is available. Moreover, in determining criterion validity, it is assumed that the criterion standard represents the "true" state of the construct of interest (17). In the current study, fall risk is supposed to be the criterion standard for balance.

Relating balance directly to falls is well-aligned with the balance construct definition and with previous studies evaluating the criterion validity of balance measures.

Balance has been defined as a person's ability not to fall (18). Which person's behaviours can be considered indicators of such a variable? A fall indicates, by definition, that a person has a decreased "ability not to fall" and hence a poor balance. Also, when a person is about to fall during a motor task, and the examiner is forced to intervene (for example, by providing physical assistance), it indicates that the "ability not to fall" is reduced, and, again, that balance is impaired.

In this regard, it is noteworthy that items from balance scales often investigate falls. For example, in some items of the Performance Oriented Mobility Assessment – Balance (POMAB), the Berg balance scale and the Mini-BESTest (likely the most used balance scales), the lowest balance level (i.e. score 0) is indicated by a fall or a near-fall (19).

This is the case of items 4, 5 and 6 of the Mini-BESTest, the "stepping correction" items, in which the subjects score 0 if they "would fall if not caught or falls spontaneously". Items with a similar scoring structure can be found in the POMAB (e.g. item 6) and the Berg balance scale (e.g. item 8).

If it is considered as safe mobility (18), i.e. the ability to reach an upright position, stand still and move in an upright position without falling, it seems natural to think of balance as linked to the risk of falling.

Indeed, a causative link between poor balance and the risk of falling has been repeatedly pointed out. The improvement obtained with balance training effectively reduces the risk of falling, a finding confirmed in systematic reviews and meta-analyses (20). Albeit not all falls occur because of a balance problem (e.g. they could occur because of footwear and home hazards (21) even with intact balance), and reduced balance does not necessarily lead to falling, it is accepted that an impaired balance implies an increased risk of falling (22).

Finally, the current work is in line with numerous studies in which the criterion validity of other balance tests has been evaluated by testing their ability to predict falls (e.g.: (19,23,24)). Some authors even overtly stated that a measure of balance that accurately identifies individuals prone to falling has predictive (i.e. criterion) validity (25).

## Different criteria for the same variable: is this a contradiction?

In our previous studies (12,15), in which we investigated the criterion validity of several ITUG measures, the Mini-BESTest was set as the criterion standard. In these studies, we concluded that measures from the sit-to-walk and turning phases of the TUG had satisfactory criterion validity for balance assessment.

Two aspects should be mentioned here in this regard.

First, the Mini-BESTest has been previously used as the criterion standard of balance, while here, it is not the criterion anymore. The fact that the Mini-BESTest criterion validity has been tested here (in a sense, it has been questioned) does not invalidate the previous investigations.

Strictly adhering to the balance definition discussed above, falls are the most reasonable indicators of balance. However, different measures (e.g. the Mini-BESTest, the turning duration, the number of falls) can all have validity for the same latent variable (e.g. balance) even if with a different validity level. The current work highlights this.

It seems more appropriate to think of validity as a gradient rather than an on-off measures property. Measures could be ranked according to their validity level, obtaining a "validity pyramid". If the balance is the ability not to fall, recording the number of falls is likely at the top of the pyramid. However, the current study highlights that when the Mini-BESTest is chosen as a balance criterion (as we previously did), this criterion is just next to the top.

Choosing a balance scale with strong psychometric characteristics, such as the Mini-BESTest, as the criterion standard for balance when recording falls is impracticable, is a reasonable solution already implemented in validity studies (e.g. (26)).

The second aspect worth discussing is that the sit-to-walk duration showed no criterion validity for balance when falls were used as the criterion standard. However, sit-to-walk measures were valid balance indicators when the balance criterion standard was the Mini-BESTest (12). Therefore, it needs to be explained why there is a relationship between sit-to-walk and the Mini-BESTest but not between sit-to-walk and the risk of falling.

In this regard, it must be stressed that different measures from the sit-to-walk phase have been assessed in the current and previous studies (12). We preferred to test the sit-to-walk duration here since this measure is more straightforward, especially in a clinical context. Its "face" validity is high, especially when compared to other sit-to-walk parameters (e.g. root mean square of angular velocity about the vertical axis during the sit-to-walk transition (12)).

In addition, it is plausible that the ITUG parameters and the Mini-BESTest do not reflect balance only.

Sit-to-stand, which is a part of the sit-to-walk task, is included in balance scales (e.g. Mini-BESTest, POMAB), but it is also a transfers component (such as transfers from bed to a wheelchair, transfers on and off the toilet) that can be found in rating scales assessing independence (e.g. FIM motor scale).

Hence, the same item can point to different latent variables (e.g. balance, independence). This reasoning suggests that the correlation between sit-to-walk measures and the Mini-BESTest is not *via* the balance but *via* the independence construct. In this regard, some multidimensionality of the Mini-BESTest has been reported (27).

## Assessing validity

Regarding the procedure for validity assessment, validation requires formulating and testing hypotheses (17). The methodology followed in the current work fully complies with this prescription.

In statistical terms, it can be said that three "null" hypotheses were tested.

1. Balance or gait measures *are not* significant predictors in multiple logistic regression models, including fall risk factors from the medical history.
2. The Akaike Information Criterion (AIC) of mobility models is *comparable to or larger* than the AIC of the simpler model, which includes only fall risk factors from the medical history.
3. Balance or gait measures *are not* selected among the predictors of the optimal model for faller risk prediction returned by the LASSO procedure.

It was assumed that if all the three null hypotheses above were rejected for one of the mobility measures tested here, this measure had satisfactory criterion validity for fall assessment.

The criteria followed in the current work for validity assessment are very stringent. However, if, as discussed previously, validity is considered a continuum, TUG duration and the angular velocity during turning also showed some criterion validity. These mobility measures were significant predictors, the AIC of their models was smaller than the one of the reference model, and this AIC difference was > 2.

The measures with the highest criterion validity as balance measures were the Mini-BESTest and the duration of the turn phase. However, even if provisional, the secondary analysis suggests that the Mini-BESTest has even higher criterion validity than the turning duration. This conclusion is substantiated by the finding that, while the LASSO logistic regression selected both mobility measures as optimal faller predictors, only the Mini-BESTest measure was included among the optimal predictors of recurrent faller status.

Regarding the statistical tests used here in the validity assessment procedure, the ROC curve and the sensitivity and specificity analysis are often used to investigate predictive criterion validity (e.g.: (23)). In addition to these rather conventional analyses, the AIC and the LASSO regression were also used.

In practical terms, the AIC is particularly suitable for selecting the most parsimonious model when multiple working hypotheses are tested. That is what was done here, where the prediction ability of a set of h+ models was evaluated. Another strength of the AIC is that it provides a simple method to rank the different hypotheses (models) from best to worst (28).

The LASSO regression, which has points of similarity with the AIC, has been chosen for analysing the current dataset because of two strengths.

First, LASSO regression performs better than standard regression when the number of events is low compared to the number of predictors. In this case, penalised regressions can provide better predictions (29).

When logistic regression is used, at least ten events per variable are commonly recommended (29). The sample size for the primary analysis, in which the single mobility measures are added to the fall risk factors from the medical history one at a time, was carefully chosen according to this rule of thumb.

Compared to the predictors' number, the number of events is relatively low when all the potential fall predictors (i.e. the fall risk factors from the medical history, the Mini-BESTest and the TUG mobility measures) are tested simultaneously in a single model (Figure 2A). This limitation is especially true for the model predicting the chance of being a recurrent faller (Figure 2B). In this regard, penalised regressions (LASSO regression included) have been reported to be effective even in the case of fewer than ten events per variable (29).

Alternatives to the LASSO regression in the case of relatively few events consist of variable selection methods based on hypothesis testing, such as forward stepwise regression. However, these approaches have serious drawbacks (29) and are harshly condemned nowadays (30).

Second, the LASSO regression also handles multicollinearity better than ordinary regression. Standard regression is unsuitable, actually, in the presence of multicollinearity. Multicollinear measures are frequent in human movement studies (e.g. (15)). For example, an obvious correlation in the dataset reported here is between the duration of the turn phase and the peak angular velocity during turning.

Finally, it is also noteworthy that LASSO regression is a recommended regression method in the TRIPOD checklist for developing and validating prediction models (31).

## The Akaike Information Criterion for model selection.

The Akaike Information Criterion (AIC) has deep theoretical (and philosophical) foundations. It moves from the concepts of entropy, Kullback-Leibler information, and likelihood theory (28).

The use of the AIC for model selection (as done in the current study) rests on the axiom that there is no "true" model but that all models are only approximations of an unknown reality or truth. Model selection in the analysis of empirical data consists in finding the model that best approximates reality given the data at hand (28).

The Kullback-Leibler information could be used for this aim since this represents the information lost when a model is used to approximate reality. In other words, the Kullback-Leibler information can be conceptualised as the distance between the model under examination and reality (32). However, since the reality is unknown, the Kullback-Leibler information is not of help in data analysis (28).

In aid intervenes the AIC, which is actually an estimate of the expected Kullback-Leibler information (28). The AIC is calculated for each competing model, and the model with the smallest AIC value is the "best". The reality remains unknown, but the model with the smallest AIC is the closest. The model with the smallest AIC is considered the best since it minimises information loss in the Kullback-Leibler sense (32).

The Least Absolute Shrinkage and Selection Operator (LASSO) regression has touchpoints with the AIC. Consider that the LASSO method (typically) optimises the deviance and that the AIC is the deviance minus (twice) the model's number of parameters (32).

Moreover, similarly to the AIC, the LASSO entirely agrees with the principle of parsimony. The coefficients of weak predictors are shrunk to zero; thus, the LASSO method "shaves away all but what is necessary" (28).

# Supplementary References

1. Franchignoni F, Horak F, Godi M, Nardone A, Giordano A. Using psychometric techniques to improve the Balance Evaluation Systems Test: the mini-BESTest. *J Rehabil Med* (2010) 42:323–331. doi: 10.2340/16501977-0537

2. Franchignoni F, Godi M, Guglielmetti S, Nardone A, Giordano A. Enhancing the usefulness of the Mini-BESTest for measuring dynamic balance: a Rasch validation study. *Eur J Phys Rehabil Med* (2015) 51:429–37.

3. Tesio L, Scarano S, Hassan S, Kumbhare D, Caronni A. WHY QUESTIONNAIRE SCORES ARE NOT MEASURES: A QUESTION-RAISING ARTICLE. *Am J Phys Med Rehabil* (2022) doi: 10.1097/PHM.0000000000002028

4. Tesio L, Caronni A, Kumbhare D, Scarano S. Interpreting results from Rasch analysis 1. The “most likely” measures coming from the model. *Disabil Rehabil* (2023)1–13. doi: 10.1080/09638288.2023.2169771

5. Tesio L, Caronni A, Simone A, Kumbhare D, Scarano S. Interpreting results from Rasch analysis 2. Advanced model applications and the data-model fit assessment. *Disabil Rehabil* (2023)1–14. doi: 10.1080/09638288.2023.2169772

6. Linacre JM, Heinemann AW, Wright BD, Granger CV, Hamilton BB. The structure and stability of the Functional Independence Measure. *Archives of physical medicine and rehabilitation* (1994) 75:127–132.

7. Studenski S, Perera S, Patel K, Rosano C, Faulkner K, Inzitari M, Brach J, Chandler J, Cawthon P, Connor EB, et al. Gait speed and survival in older adults. *JAMA* (2011) 305:50–58. doi: 10.1001/jama.2010.1923

8. Rota V, Perucca L, Simone A, Tesio L. Walk ratio (step length/cadence) as a summary index of neuromotor control of gait: application to multiple sclerosis. *International Journal of Rehabilitation Research* (2011) 34:265. doi: 10.1097/MRR.0b013e328347be02

9. Podsiadlo D, Richardson S. The timed “Up & Go”: a test of basic functional mobility for frail elderly persons. *J Am Geriatr Soc* (1991) 39:142–148. doi: 10.1111/j.1532-5415.1991.tb01616.x

10. Caronni A, Picardi M, Pintavalle G, Aristidou E, Redaelli V, Antoniotti P, Sterpi I, Tropea P, Corbo M. Responsiveness to rehabilitation of balance and gait impairment in elderly with peripheral neuropathy. *Journal of biomechanics* (2019) 94:31–38.

11. Mellone S, Tacconi C, Chiari L. Validity of a Smartphone-based instrumented Timed Up and Go. *Gait Posture* (2012) 36:163–165. doi: 10.1016/j.gaitpost.2012.02.006

12. Picardi M, Redaelli V, Antoniotti P, Pintavalle G, Aristidou E, Sterpi I, Meloni M, Corbo M, Caronni A. Turning and sit-to-walk measures from the instrumented Timed Up and Go test return valid and responsive measures of dynamic balance in Parkinson’s disease. *Clin Biomech (Bristol, Avon)* (2020) 80:105177. doi: 10.1016/j.clinbiomech.2020.105177

13. Tibshirani R. Regression shrinkage and selection via the lasso. *Journal of the Royal Statistical Society: Series B (Methodological)* (1996) 58:267–288.

14. Ranstam J, Cook JA. LASSO regression. *BJS (British Journal of Surgery)* (2018) 105:1348–1348. doi: 10.1002/bjs.10895

15. Caronni A, Sterpi I, Antoniotti P, Aristidou E, Nicolaci F, Picardi M, Pintavalle G, Redaelli V, Achille G, Sciumè L, et al. Criterion validity of the instrumented Timed Up and Go test: A partial least square regression study. *Gait Posture* (2018) 61:287–293. doi: 10.1016/j.gaitpost.2018.01.015

16. Friedman J, Hastie T, Tibshirani R. Regularization Paths for Generalized Linear Models via Coordinate Descent. *Journal of Statistical Software* (2010) 33:1–22. doi: 10.18637/jss.v033.i01

17. de Vet HCW, Terwee CB, Mokkink LB, Knol DL. “Validity.,” *Measurement in Medicine: A Practical Guide*. Practical Guides to Biostatistics and Epidemiology. Cambridge University Press (2011). p. 150–201 doi: 10.1017/CBO9780511996214.007

18. Pollock AS, Durward BR, Rowe PJ, Paul JP. What is balance? *Clin Rehabil* (2000) 14:402–406. doi: 10.1191/0269215500cr342oa

19. Shumway-Cook A, Woollacott MH. *Motor Control: Translating Research Into Clinical Practice*. Wolters Kluwer, Lippincott Williams & Wilkins (2012). https://books.google.it/books?id=vwofkgAACAAJ

20. Kannus P, Sievänen H, Palvanen M, Järvinen T, Parkkari J. Prevention of falls and consequent injuries in elderly people. *The Lancet* (2005) 366:1885–1893.

21. Lamb SE, Bruce J, Hossain A, Ji C, Longo R, Lall R, Bojke C, Hulme C, Withers E, Finnegan S, et al. Screening and Intervention to Prevent Falls and Fractures in Older People. *N Engl J Med* (2020) 383:1848–1859. doi: 10.1056/NEJMoa2001500

22. Korhonen N, Niemi S, Parkkari J, Sievänen H, Kannus P. Incidence of fall-related traumatic brain injuries among older Finnish adults between 1970 and 2011. *JAMA* (2013) 309:1891–1892. doi: 10.1001/jama.2013.3356

23. Raîche M, Hébert R, Prince F, Corriveau H. Screening older adults at risk of falling with the Tinetti balance scale. *Lancet* (2000) 356:1001–1002. doi: 10.1016/S0140-6736(00)02695-7

24. Duncan PW, Studenski S, Chandler J, Prescott B. Functional reach: predictive validity in a sample of elderly male veterans. *J Gerontol* (1992) 47:M93-98. doi: 10.1093/geronj/47.3.m93

25. Berg K. Balance and its measure in the elderly: a review. *Physiotherapy Canada* (1989) 41:240–246. doi: 10.3138/ptc.41.5.240

26. Hofheinz M, Schusterschitz C. Dual task interference in estimating the risk of falls and measuring change: a comparative, psychometric study of four measurements. *Clin Rehabil* (2010) 24:831–842. doi: 10.1177/0269215510367993

27. Benka Wallén M, Sorjonen K, Löfgren N, Franzén E. Structural Validity of the Mini-Balance Evaluation Systems Test (Mini-BESTest) in People With Mild to Moderate Parkinson Disease. *Phys Ther* (2016) 96:1799–1806. doi: 10.2522/ptj.20150334

28. Burnham KP, Anderson DR. Kullback-Leibler information as a basis for strong inference in ecological studies. *Wildl Res* (2001) 28:111–119. doi: 10.1071/wr99107

29. Pavlou M, Ambler G, Seaman SR, Guttmann O, Elliott P, King M, Omar RZ. How to develop a more accurate risk prediction model when there are few events. *BMJ* (2015) 351:h3868. doi: 10.1136/bmj.h3868

30. Harrell FE. “Multivariable Modeling Strategies.,” *Regression modeling strategies with applications to linear models, logistic and ordinal regression, and survival analysis*. Spinger (2015)

31. Collins GS, Reitsma JB, Altman DG, Moons KGM. Transparent reporting of a multivariable prediction model for individual prognosis or diagnosis (TRIPOD): the TRIPOD statement. *BMJ* (2015) 350:g7594. doi: 10.1136/bmj.g7594

32. Burnham KP, Anderson DR, Huyvaert KP. AIC model selection and multimodel inference in behavioral ecology: some background, observations, and comparisons. *Behav Ecol Sociobiol* (2011) 65:23–35. doi: 10.1007/s00265-010-1029-6
